# Seasonal patterns in psychiatric emergency care visits

**DOI:** 10.1192/j.eurpsy.2021.1876

**Published:** 2021-08-13

**Authors:** D. Hernández-Calle, M.F. Bravo-Ortiz

**Affiliations:** Psychiatry, Hospital Universitario La Paz, Madrid, Spain

**Keywords:** Emergency Care

## Abstract

**Introduction:**

Psychiatric emergency visits have with associated with seasonal pattern that reflect psychosocial factors. Its knowledge could proving valuable insight about help seeking behaviour of patiens suffering with mental illness.

**Objectives:**

Our aim was to analyse weekly and monthly seasonality

**Methods:**

Daily urgency visits were extracted from electronic medical records of Hospital Universitario La Paz from 1st January 2019 to 31st December 2019. A poisson multivariate model was performed with day of the week and month as covariates. Predictive margins were estimated

**Results:**

Psychiatric emergency visits were less frequent in Saturday and Sunday (5.5 visits per day) than weekdays (7.5 visits per day). Not differences were observed among months.

**Conclusions:**

A weekly season pattern was observed with less psychiatric emergency visits during weekends.

**Disclosure:**

No significant relationships.

